# Cerebral Syphilitic Gumma in Immunocompetent Man, Japan

**DOI:** 10.3201/eid2402.171444

**Published:** 2018-02

**Authors:** Tatsuya Kodama, Hidenori Sato, Morichika Osa, Yuji Fujikura, Akihiko Kawana

**Affiliations:** National Defense Medical College, Saitama, Japan (T. Kodama, M. Osa, Y. Fujikura, A. Kawana);; IMSUT Hospital, The Institute of Medical Science, The University of Tokyo, Tokyo, Japan (H. Sato)

**Keywords:** syphilis, syphilitic gumma, neurosyphilis, Japan, sexually transmitted infections, bacteria, cerebral syphilitic gumma

## Abstract

Although cerebral syphilitic gummas are generally considered to be rare manifestations of tertiary syphilis, many reports exist of early cerebral syphilitic gumma. Our finding of cerebral syphilitic gumma in an HIV-negative man within 5 months after syphilis infection suggests that this condition should be considered in syphilis patients who have neurologic symptoms.

In September 2017, a 36-year-old man sought care at the National Defense Medical College Hospital (Saitama, Japan) because of hearing loss in his right ear and right-sided facial weakness, which had been worsening for 2 weeks. Other than heterosexual intercourse with a commercial sex worker 5 months earlier and a broken right fibula 3 months earlier, his medical history was unremarkable. At a routine medical examination 5 months earlier, serum rapid plasma reagin (RPR) and treponema pallidum hemagglutination (TPHA) were negative. However, TPHA was positive (1:145.3) at a preoperative workup for his right fibula fracture.

At hospital admission, his temperature was 36.4°C; pulse and respiratory rates were normal. Physical examination revealed right-side facial paralysis. Ophthalmologic findings were normal. Meningeal signs were absent, and deep sensation was intact. No skin lesions were apparent. Audiograms revealed severe-to-profound right sensorineural hearing loss. Brain computed tomography scan showed no abnormalities. Magnetic resonance imaging (MRI) showed a nodule-like lesion in the left temporal lobe, which was enhanced on T1-weighted imaging (Figure, panel A). Enhancement was also found within the cisternal segment of the vestibulocochlear nerve complex and the facial nerve on gadolinium-enhanced T1-weighted imaging (Figure, panel B). The mass-like lesion was hyperintense on T2-weighted images and fluid-attenuated inversion recovery images (Figure, panel C).

**Figure:**
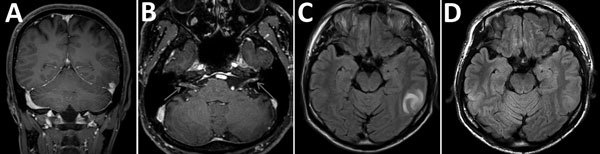
Brain magnetic resonance imaging findings in a 36-year-old immunocompetent man before (A, B, C) and after (D) treatment for cerebral syphilitic gumma, Saitama, Japan. A) Gadolinium-enhanced T1-weighted coronal image shows an enhanced nodular lesion in the left temporal lobe. B) Axial gadolinium-enhanced T1-weighted image shows enhancement within the cisternal segment of both the vestibulocochlear nerve complex and the facial nerve. C) Axial fluid-attenuated inversion recovery images show a hyperintense lesion-like mass in the left temporal lobe. D) Axial fluid-attenuated inversion recovery image shows complete resolution after discontinuation of treatment.

At admission, RPR titers were 1:5.9 (reference 1:<1) and TPHA titers were 1:327.1 (reference 1:<80). An HIV test was negative, and the patient did not have any other history or laboratory findings suggesting immunosuppression. Blood culture was negative. Cerebrospinal fluid (CSF) analysis showed 142 cells/μL (reference <5 cells/μL), of which 96% were lymphocytes; glucose level 60 mg/dL (reference range 45–80 mg/dL; serum glucose level 96 mg/dL [reference range 70–110 mg/dL]); and a total protein level of 64 mg/dL (reference 15–45 mg/dL). CSF RPR titer was 1:2.4 and treponema pallidum latex agglutination titer was 1:53.4. Fluorescent treponemal antibody absorption was 2+ positive. CSF culture was negative.

We suspected that the right facial nerve palsy and the hearing loss were due to neurosyphilis causing cranial nerve (CN) VII and CN VIII dysfunction and that the lesion on the left temporal lobe was a cerebral syphilitic gumma. However, we could not exclude primary central nervous system lymphoma or a brain tumor, such as glioma, and bacterial brain abscess.

Considering the possibility of bacterial brain abscess, we used ceftriaxone for treatment (*1*), which is also a recommended alternative regimen for treating neurosyphilis under the 2015 UK national guidelines (*2*). A few hours after the initial dose of ceftriaxone (2 g), the patient developed a Jarisch–Herxheimer reaction consisting of left temporal headache and nausea, but the symptoms resolved spontaneously in half a day. We found no evidence of bacterial hematogenous spread and bacterial parameningeal foci, which could have caused bacterial brain abscess. Therefore, we administered ceftriaxone (2 g/d for 2 weeks) as treatment for neurosyphilis. Thereafter, the right facial nerve palsy improved markedly, and the hearing loss improved gradually.

An MRI performed 2 weeks after treatment started indicated enhancement in the lesion on T2-weighted image and fluid-attenuated inversion recovery images had disappeared (Figure, panel D). The hyperintensity of the cranial nerve had also resolved. Therefore, we concluded that the lesion had indeed been a cerebral syphilitic gumma. Repeat studies of CSF RPR and serologic RPR 6 months after completion of therapy were both 1:<1. There have been no signs of recurrence as of 1 year after therapy.

The classic diagnosis of cerebral syphilitic gumma is based on the combination of several factors: the prior treatment of asymptomatic syphilis, the clinical presentation of focal seizures, positive serologic status, characteristic MRI findings, and a clinical response to penicillin (*3*). In some cases, the diagnosis is made by autopsy (*4*) or biopsy (*5*,*6*), but we did not perform a cerebral biopsy because of the degree of invasiveness and because spirochetes are rarely found in cerebral syphilitic gumma (*7*).

RPR and TPHA can become positive as late as 6 weeks after infection (*8*). Therefore, based on the results of serum RPR and TPHA 5 months and 3 months before onset, the patient probably contracted syphilis within 5 months before detection of the cerebral syphilitic gumma.

Many reports exist of early cerebral syphilitic gumma. However, in most of those cases, estimating how long patients had syphilis until cerebral syphilitic gumma appeared was based on medical interviews of history of sexual contact or specific symptoms of each stage. Recently, Tsuboi et al. (9) and Koizumi et al. (*10*) detected gumma in HIV-positive patients, with specific timing. The HIV-negative patient reported here also had early cerebral syphilitic gumma, within 5 months after syphilis infection, diagnosed accurately by confirming seronegativity. This case suggests that cerebral syphilitic gumma should be considered in patients with syphilis who have neurologic signs and symptoms.
